# Competition between Public and Private Maternity Care Providers in France: Evidence on Market Segmentation

**DOI:** 10.3390/ijerph17217846

**Published:** 2020-10-26

**Authors:** Daniel Herrera-Araujo, Lise Rochaix

**Affiliations:** 1 PSL Research University, LEDa (CGEMP) UMR CNRS [8007], Université Paris-Dauphine, UMR IRD [260], 75016 Paris, France; 2 Panthéon-Sorbonne, University of Paris 1, 75231 Paris, France; 3 Hospinnomics, Assistance Publique-Hôpitaux de Paris and Paris School of Economics, 75014 Paris, France; lise.rochaix@psemail.eu

**Keywords:** maternity units, quality differentiation, substitution, market segmentation, public-private mix

## Abstract

The purpose of this paper is to investigate the potential for segmentation in hospital markets, using the French case where private for-profit providers play an important role having nearly 25% of market shares, and where prices are regulated, leading to quality competition. Using a stylized economic model of hospital competition, we investigate the potential for displacement between vertically differentiated public and private providers, focusing on maternity units where user choice is central. Building over the model, we test the following three hypotheses. First, the number of public maternity units is likely to be much larger in less populated departments than in more populated ones. Second, as the number of public maternity units decreases, the profitability constraint should allow more private players into the market. Third, private units are closer substitutes to other private units than to public units. Building an exhaustive and nationwide data set on the activity of maternity services linked to detailed data at a hospital level, we use an event study framework, which exploits two sources of variation: (1) The variation over time in the number of maternity units and (2) the variation in users’ choices. We find support for our hypotheses, indicating that segmentation is at work in these markets with asymmetrical effects between public and private sectors that need to be accounted for when deciding on public market entry or exit.

## 1. Introduction

In developed countries, hospital care is of upmost importance because of its size relative to GDP and its impact on individuals’ well-being. In France, this industry is divided into the public and private sector, with the latter comprising both for-profit and non-for-profit hospitals. With more than 25% of activity in the hospital market, the private sector in France is quite large. It is generally acknowledged that competition increases quality [1]. Given the presence of both sectors, the degree of competition within and between sectors depends, however, on whether markets are segmented or not.

With one of the largest hospital industries worldwide and increasing expenditure in health care, the historical division between public and private providers in the French hospital sector offers unique insights on the public-private mix of health care services. Among all services, three key aspects make maternity care the most representative of this public-private mix. First, and foremost users’ preferences for public or private providers play an important role (in the following, we refer to users’ preferences and choices as a mix between the patients and their doctors). In the French healthcare system, users’ maternity choices are unrestricted. Users will choose to opt for their preferred maternity, which in turn may have an effect on the strategic interactions between public and private maternity services [2,3,4,5,6,7,8,9,10]. Second, French healthcare providers are not able to set the price for their services and mostly rely on vertical differentiation, higher quality being an important lever to attract users [11,12,13,14,15]. Third, public hospitals are required to provide basic care across the French territory and closure decisions are taken on a national and local level, usually for patient safety and risk prevention. Private exit decisions on the other hand are taken unilaterally, usually based on profitability arguments [16,17,18,19].

We use a stylized theoretical model of hospital competition [20,21,22,23] to analyze the French maternity market. The model is constructed to reflect the current French institutional setting [24], where prices are fixed, and where users may freely choose their provider. Given a fixed price system, the model suggests that the existence of quality competition depends on users’ ability to choose between maternity units. The model also suggests that users’ preferences shape the substituability between maternity units. Last, the model suggests that the number of units should be positively correlated with the number of potential users.

Drawing on the implications of our theoretical model, we test three hypotheses. Given the public mandate on equal access to healthcare across France, we argue that the public sector budget constraint is not too biding in smaller departments. Other factors calling for public provision are services whose value to the consumer cannot be entirely captured by the supplier and which are, therefore, not supplied by the private sector. For hospital care, such services include research, disease prevention, informing the public and training. These are all part of the public mandate in France, which is mandatory for all public and some of the private (non-for-profit) hospitals. We therefore hypothesize that the number of public maternity units is much larger in less populated departments than in more populated ones. Next, we argue that the number of public hospitals will have a direct impact on the ability of the private sector to operate. We hypothesize that as the number of public maternity units decreases, more private units will be able to operate in the market. Finally, we argue that users’ preferences are heterogeneous and that the patterns of substitution may differ between private and public units. We hypothesize that private units are closer substitutes to other private units than to public units. To test our hypotheses, we constructed a data set that includes an exhaustive and nationwide record on hospital activity, complemented with a publicly available database with detailed hospital-level information on financial status, heavy equipment, labor force, and among other relevant variables such as geographic location, legal status, field of activity, date of opening and closure, and other hospital level characteristics.

We elaborate on this setting to investigate to what extent public and private maternity units are substitutes by exploiting two sources of variation: (1) The variation over time in the number of maternity units, which we refer to as substitution at the extensive margin and (2) the variation in users’ choices, which we refer to as substitution at the intensive margin. We are interested in assessing whether there is a substitution on the extensive margin of maternity units between sectors, and conditional on the number of units in the market, whether there is a substitution on the intensive margin between the public and private providers.

In line with hypothesis #1, we observe a negative relationship between the birth quantiles and the number of public maternity units. We find more public maternity units in less populated departments. Next, we find evidence of substitution between public and private units across departments, which supports hypothesis #2. A higher number of public units decreases the number of private units. Our point estimates suggest that with an additional public unit, nearly 0.25 private maternity units are displaced. The point estimates of our preferred specification, however, are not precisely estimated. Our empirical approach parallels that of Berry & Waldgogel 1999 [25], who studied the links between market size and the number of firms active in the market for radio stations in the U.S. Their results suggest that public stations crowd-out private stations only in larger markets. Unlike Berry & Waldfogel (1999) [25], we find evidence of extensive margin substitution across all market sizes, consistent with an entry model with a constant fixed cost where firms capture all expected benefits.

Using a catchment area analysis, we provide evidence supporting hypothesis #3. Our results suggest that public units’ market shares decrease with the number of other public units in the same market but are not affected by the number of private units. Similarly, private units’ market shares decrease with the number of other private units but are also affected by the number of other public units.

To the best of our knowledge, there is only one empirical paper, by Cooper et al. (2018) [26], which specifically deals with public/private hospital competition. The paper focuses on private hospital entry in the UK market, yet only considers the intensive margin, i.e., quality, and not the extensive margin, i.e., actual entry. In the insurance literature, Cutler (1996) [27] analyses whether public insurance crowds-out private insurance, exploiting the expansion of Medicaid between 1987 and 1990. He found that around 50% of the increase in Medicaid coverage was associated with a reduction in private insurance coverage at the expense of public insurance. Similarly, there has been a great deal of interest in recent years in competition between private and public providers of education, but evidence is also quite thin [28,29,30]. Our paper on hospital service segmentation in France seeks to fill this gap in the literature.

The paper is organized as follows: Section 2 describes the French maternity units’ market; Section 3 presents a theoretical model that captures key trade-offs in French maternity units’ market; Section 4 presents the data and some descriptive evidence on the relationship between public and private maternity units of different sizes; Section 5 presents the empirical strategy and the results; and Section 6 discusses and concludes the discussed information.

## 2. Market Characteristics

The French private for-profit sector is one of the largest in France, compared to other countries, including the U.S. The only other country to which it can be compared in this respect is Germany where it is slightly higher (30%). In France, in 2018, 62% of hospital beds were public, 14% were private not-for-profit, and 24% were private for-profit. But unlike France, it is only since 2000 that Germany has engaged in a large movement of privatization of hospital services as Länders, in charge of funding hospital infrastructures, try to reduce their financial debts by shifting to private for-profit hospitals. In France, the maternity market is a prime example of the private-public mix.

To operate, a maternity unit is required to satisfy several constraints. All hospitals with maternity units have an obligation of providing 24/7 consultant-level obstetrician services, either on site, for units with more than 1500 births, or on call, and available within 20 min for units with less than 1500 births. All maternity units are required to have 24/7 midwife presence. These requirements introduce significant fixed and variable costs, leading to a potential decrease in the profitability of maternity units.

French maternity units are divided into a three level risk-tiered system: Level 1 maternity units handle pregnancies with no particular risk factors, which represent the largest number of births; level 2 maternity units offer neonatal intensive care and treatment for premature infants from the 33rd week of pregnancy; and maternity units of level 3 specialize in births with severe complications, including extreme premature births, high risk pregnancies, and provide newborn resuscitation. All units are organized in networks centered on level 3 units, with clear transfer protocols and network arrangements in case of unforeseen complications. Hence, French maternity units are not only differentiated by their ownership status but also their respective responsibilities, with transfers organized according to severity within the network. Unfortunately, we are not able to observe these transfers across maternity units. As we describe in detail below, most of the identifying variation comes from units of level 1. For this reason, we concentrate all of our analyses on the interactions between private/public ownership status and do not differentiate between levels.

The market for maternity care in France has experienced changes in the past few decades, with a steady reduction in the number of maternity units, from +600 in the early 2000 to +500 today. Safety regulations which were introduced in 1972, 1988, and more recently, in 1998 are partly accountable for this reduction. Recent exits of private for-profit maternity units are mostly explained by a payment reform: The adoption from 2004 onward of a Diagnosis Related Group (DRG) hospital funding scheme. Since then, every hospital, public or private, is funded according to a nationally fixed tariff, set for each DRG. Before 2004, the private sector was mainly funded through regionally negotiated tariffs, while the public sector was funded by a global budget. As additional patients now represent additional revenues, the DRG system introduces high-powered activity incentives for public hospitals. During our period of analysis, from 2009 to 2014, all public hospitals in France were under this DRG payment scheme [8]. Given the intensity of the changes, it calls for an analysis of competition in the French hospital market.

Another key feature of the French case is that it offers its users freedom of choice between public or private maternity units and out-of-pocket payments play a small role. As child birth costs are mostly covered by the national health insurance, price differences play a minor role in maternity unit choice. Extra billing for a particular physician or a single room will be refunded by supplementary health insurance. Almost 90% of men and women at a working age are covered by voluntary private health insurance, in addition to the coverage provided by the national health insurance system. Out-of-pocket payments are quite low. This implies that non-price attributes, such as proximity, perceived safety, or staff responsiveness play a prominent role in a user’s maternity unit choice.

## 3. The Theoretical Model

Let quality of hospital *j* be denoted by, $z_{j}$. It corresponds to a uni-dimension variable and has a vertical dimension, i.e., “more is better”. Let $q_{j}$ denote demand for hospital *j*. It is given by,

(1) $$\begin{array}{c} q_{j}=\sum_{k\in \{pub,pri\}}s_{j}^{k}\left(z_{j},Z_{-j}\right)D^{k}\left(\bar{p},z_{j},Z_{-j}\right), \end{array}$$

where, $s_{j}^{k}$ is the market share for hospital *j* from patients that prefer sector k∈{pub,pri}, $D^{k}$ is the market demand from these type of patients, $\bar{p}$ is the fixed price *j*, and $Z_{-j}$ is the quality for all hospitals except *j*. Note that the demand for hospital *j* depends on the size of the market for each sector, which can be allowed to overlap.

Next, assume all hospitals use the same technology and face the same input prices. Let their costs be described by:

(2) $$\begin{array}{c} c_{j}=c\left(q_{j},z_{j}\right)+F. \end{array}$$

where, $c(q_{j},z_{j})$ is a variable cost and $F_{j}$ is a fixed cost of entry. Thus, the profit of the hospital is given by:

(3) $$\begin{array}{c} \pi_{j}=\bar{p}q_{j}-c\left(q_{j},z_{j}\right)-F_{j}. \end{array}$$

Assuming a Nash–Bertrand solution concept, the equilibrium qualities $z^{*}$ are characterized by solutions to the following equations across all hospitals *j*:

(4) $$\begin{array}{c} \frac{\partial \pi_{j}}{\partial z_{j}}=\sum_{k=\{pub,pri\}}\left[\bar{p}-\frac{\partial c_{j}}{\partial q_{j}}\right]\left\{\frac{\partial s_{j}^{k}}{\partial z_{j}}D^{k}\left(\bar{p},z_{j},Z_{-j}\right)+s_{j}^{k}\frac{\partial D^{k}\left(\bar{p},z_{j},Z_{-j}\right)}{\partial z_{j}}\right\}-\frac{\partial c_{j}}{\partial z_{j}}=0, \end{array}$$

where $\frac{\partial s_{j}^{k}}{\partial z_{j}}$ is a business stealing effect, while $\frac{\partial D^{k}}{\partial z_{j}}$ is a market expansion effect. Furthermore, assume that there is free entry and exit, so that all hospitals earn zero profits in equilibrium.

(5) $$\begin{array}{c} \pi_{j}^{*}\left(n_{pub}^{*},n_{pri}^{*}\right)=\bar{p}q_{j}^{*}-c\left(q_{j}^{*},z_{j}^{*}\right)-F_{j}=0. \end{array}$$

This last equilibrium condition rationalizes the fact that the number of hospitals in a market is limited to a finite number. Moreover, it also posits that the number of private and public hospitals is endogenous and depends on the profitability condition. In light of our theoretical model, we offer several testable hypotheses using this simple framework:

1 As we expect that the profitability condition is not too binding for the public sector, the number of public maternity units should be much larger in less populated departments than in more populated ones;
2 As the number of public maternity units decreases, the profitability constraint should allow more players in the market. We hypothesize that more private units will be able to operate in the market;
3 Finally, we hypothesize that private units are closer substitutes to other private units than public units.

## 4. Data

To consistently estimate the competitive effects of public versus private maternity units, we require detailed information on the number, location, level, and activity of each unit. We construct a merged database on obstetric services available for public and private hospitals in France. The following section describes the different data sources linked to construct this unique database.

### 4.1. Data Sources

Our main source of information is an extraction from the Programme de médicalisation des systèmes d’information, (PMSI), a publicly available administrative database providing an exhaustive and nationwide record on hospital activity. ScanSanté is based on a DRG-classification of activities, covering all public and private hospitals. It provides data on all claims paid by national health insurance to hospitals and is therefore the main source of information on hospital activity and associated expenditure. We used information on the total number of clinical procedures filed from 2009 until 2014. We supplemented our data with the number of patient standardized discharge records per unit-DRG and their residence zip-code.

For information on a maternity unit level, we used Hospidiag, a publicly available meta-database gathering detailed hospital-level information on activity, financial status, heavy equipment, labor force, among other relevant variables. As hospital characteristics vary over time (i.e., the number of mid-wives per obstetrician or C-section rates may change over the course of a couple of years), controlling for such time-varying characteristics is necessary to avoid potential confounding effects.

We supplemented our main data source with variables from FINESS database (FINESS stands for Fichier National des Etablissements Sanitaires et Sociaux), which is a national directory of health and social establishments maintained by the Regional Department of Health and Social Affairs and the Departmental Directorate for Health and Social Affairs (in French it is the Direction régionale des affaires sanitaires et sociales and Direction Départementale des Affaires Sanitaires et Sociales, respectively). Each FINESS identifier is paired with data on the hospital’s geographic location, legal status, field of activity, date of opening and closure, and other hospital level characteristics.

### 4.2. Summary Statistics

To capture general tendencies, Table 1 reports summary statistics for maternity units in France from 2009 to 2014. There is substantial variation in the number of births across departments, with an average number of births per department of around 13,000 births, ranging from 500 births to 56,000 births. On a department level, we see that maternity unit market shares also display substantial variation. All analysis was done using Stata MP 16.

On average, the length of stay in a maternity unit follows clinical guidelines. The ratio between the number of days spent in a maternity unit and the number of days recommended by clinical guideline is 1. Users have a 20% probability of having a C-section and a 75% probability of having an epidural. There are around 50 births per obstetrician, while there are 4 mid-wives per obstetrician. The low number of births per obstetrician may be related with the fact births are taken care of by a team composed of obstetricians and mid-wives. The bed utilization ratio is close to 60% (a rate higher than 100% implies that beds assigned to the obstetric department are used for other conditions), and the average number of beds is close to 42. Regarding technical equipment, hospitals and clinics that include a maternity unit service have on average a number of scanners and MRIs of 1.8 and 1.9, respectively.

The public sector has, on average, the largest number of maternity units per department (4.4), compared to the for-profit sector (2.6) and the non-for-profit sector (0.5). Most of the public units are level 2, while most of the for-profit and non-for-profit units are level 1. Changes over time in the number of maternity units and in their level are reported in Table 2. We observed an overall decrease in the number of level 1 units across all sectors, with a stronger effect for the private sector. This results from either unit closures or, to a lesser extent, changes in unit level for the for-profit sector (no level changes occurred for non-for-profit units over the study period).

**Figure 1:**
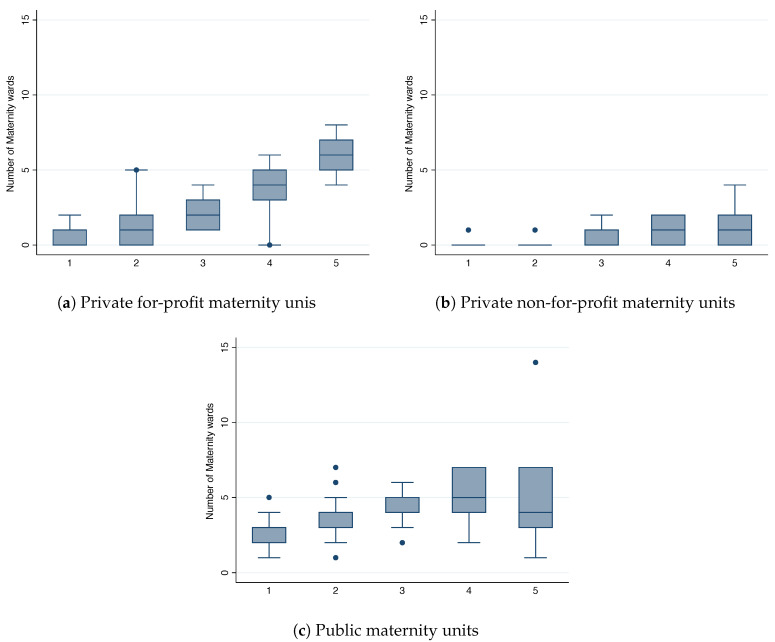
Number of maternity units, by birth quantile.

An analysis of the distribution pattern of private and public maternity units across departments in France allows an assessment of the extent to which the number of units correlate with the demand for maternity care. We expect that in smaller markets, public units are the main providers of maternity care because private maternity units cannot profitably sustain activity. In larger markets, public and private maternity units are likely to be rivals. Figure 1 depicts the relationship between market size per birth quantile, as measured by the number of births by department, and number of private, non-for-profit and public units, using a box-and-whiskers plot. This plot gives a simple descriptive and “non-parametric” fit to the distribution of maternity units. As Figure 1 shows, the number of for-profit, non-for-profit and public maternity units increases with the size of the market. The fact that the number of maternity units is positively related to market size is not surprising. As users care about how distant maternity units are from their homes, this naturally generates geographically separate markets. As the cities/departments grow in population size, say by adding new neighborhoods, maternity units too far away from a mother’s home will not be part of the considered set. Hence, we expect that the number of maternity units will have a linear relationship to the market size. Finally, the average driving time between the centroid of a zip-code and a maternity is 22 min.

**Figure 2:**
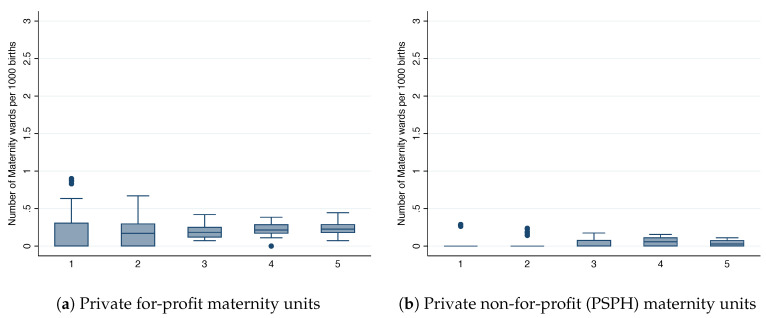

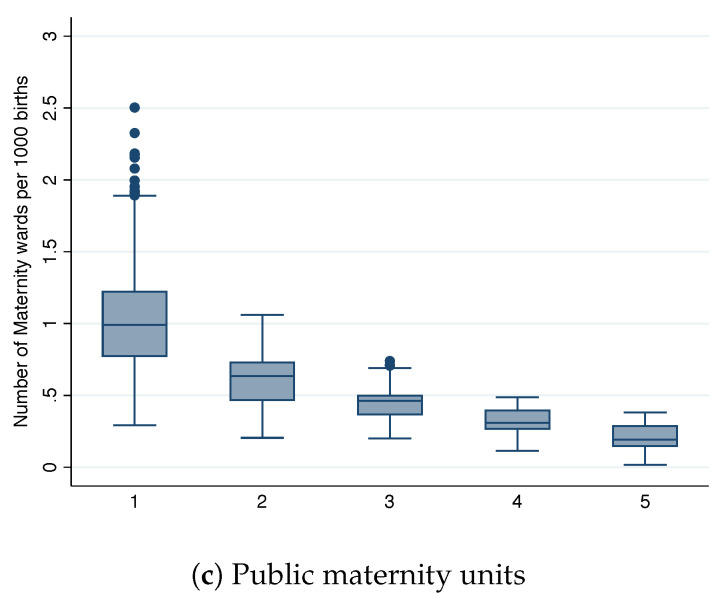
Number of maternity units per 1000 births, by birth quantile.

Figure 2 depicts the relationship between market size per birth quantile and the number of units per 1000 births. In line with the idea of geographically segmented markets, we observe a nearly constant number of maternity units across all market sizes per birth quantiles for the private sector. A maternity unit in the private for-profit sector seems to require around 3000 births for sustainability. In line with hypothesis #1, we observe a negative relationship between the birth quantiles and the number of public maternity units. The implied number of births needed to sustain a public maternity unit is equal to 1000 births in the first birth quantile, and it converges to the private sector number of births in the fifth birth quantile. The fact that public maternity units can operate with a lower number of births than the private sector suggests that they might be incurring financial losses. It might also be the case that publicly operated maternity units are less expensive than private units, although it has not been documented. Moreover, the locations where public units are operating at a loss correspond to departments where under-provision is more likely to occur.

## 5. Empirical Strategy

In order to investigate the structure of the French market for maternity units, we first analyze the extent to which private maternity units compete with other private units (for-profit and non-for-profit) or with public maternity units on an extensive margin in the same market, using a reduced form approach. Next, we analyze the intensive margin using a reduced-form analysis to assess the substituability between maternity units.

### 5.1. Extensive Margin Substitution Analysis

In this subsection, we introduce the reduced-form analysis on extensive margin substitution between private and public hospitals at an aggregate level. We first define the relevant market to then provide the empirical strategy followed by the results.

#### 5.1.1. Market Definition

As the extensive margin analysis examines the correlation between the number of public and private units, it requires the markets to be fixed. For this, we define the relevant markets of maternity units using department borders, which results in 94 markets that vary in population size. That is, for the extensive margin analysis, we define a market to be a department-year. The departments are further divided into quantiles according to their population size to examine differences between market sizes. Within each department, we aggregate activity between private and public sectors.

A general concern related to market definition is the size of the market. Too-large market definitions will include too many competitors and hence the effect of competition will be underestimated. Too-small market definitions, on the other hand, will overestimate the relation because less direct competitors are excluded. French departments are relatively large and hence there is the possibility of relatively high differences between travel times to different maternity units. Considering this, we assume that our strategy is more likely to identify too many units as potential competitors and therefore underestimate the substituability between units that are effectively competing for users. Furthermore, using department boundaries as markets may introduce a bias through competitors that are located close to a unit, but in a different department.

#### 5.1.2. Empirical Strategy

We wish to assess whether there is substitution on the extensive margin between public and private activity. In line with our theoretical model, for the private sector to enter and stay in the market, the demand for maternity services must be high enough so that the revenue covers the fixed costs of providing care. However, the public sector might operate incurring losses primarily to fulfill its public mandate to the community it serves.

To investigate whether substitution on the extensive margin is occurring between private and public units, we regress the number of maternity units in a market on its market determinants along with the number of competitors from a different sector in the same market. We interact the number of competing units from a different sector with birth quantiles dummies to assess the competitive impact on varying market sizes. We use the following specification:

(6) $$\begin{array}{cc} FP_{mt} & =\kappa_{1}+\sum_{k=1}^{5}\beta_{1k}*PU_{mt}*BQ_{mt}^{k}+\sum_{k=1}^{5}\beta_{2k}*NFP_{mt}*BQ_{mt}^{k}+\gamma_{m}+\gamma_{t}+\epsilon_{mt}^{1}, \end{array}$$

where $FP_{mt}$ and $PU_{mt}$ represent the number of maternity units in the market *m*, at period *t*, respectively for for-profit units and public units; ${\mathrm{BQ}}_{mt}^{k}$ are dummy variables equal to 1 when market *m* at period *t* is among the *j*’s quantile in terms of number of births per market across all markets in France; and $\gamma_{m}$ and $\gamma_{t}$ are department and time fixed effects, respectively.

As we control for department fixed effects in these regressions, our identifying variation comes from two different sources: (1) The exit of public maternity units across the different birth quantiles and (2) the variation in the number of births, which shifts the birth quantile ranking across the markets. As the ranking across birth quantiles is fairly constant across departments, the most important source of variation comes from the variation in the number of units. To the extent that assuming that unobservable characteristics (for example, tastes for public/private maternity units) do not change over time might be a strong assumption, our results are intended to present correlations rather than a causal impact. Although not reported, we performed robustness checks using one-year lags for the explanatory variables. The results are robust to this alternative specification.

To try to assess the extent of bias, we report results from an instrumental variable approach. As an instrument, we proxy the financial status of public maternity units within a department. A public hospital with financial issues may be forced to close some of its services, leading to the closure of public maternity units. For this, we use three different proxies: Gross profit rate, gross internal financing capacity, and net internal financing capacity. All attempt to capture the hospital’s ability to finance its own current expenses and current and future investments. The difference between the gross and net internal financing capacity is that the net accounts for reimbursements of current investments. The decaying financial status of public hospitals within a department may force some public hospitals to consider closing some of its maternity units and re-organize the maternity network within the department. The identifying assumption is that the average financial status of public maternity units is not correlated with changes in observable characteristics such as changes in users’ tastes for public/private maternity units.

#### 5.1.3. Results on Extensive Margin Substitution

In Table 3, we report the estimated coefficients for the extensive margin substitution analysis, estimated separately for fixed effects (columns (1) and (2)), and fixed effects with the instrumental variable approach (columns (3) and (4)). An observation is a department in one year. The dependent variable in all regressions is the number of maternity units in the private sector in the department. Independent variables are the number of units, and the number of units interacted with birth quantile dummies. Although not reported for simplicity’s sake, the number of private non-for-profit units, interacted with birth quantile dummies, is used in both regressions. Department-fixed effects, time-fixed effects, and the number of births per department are also included in the regressions.

Looking at the number of all private maternity units per department (column (1)), our results indicate that there is evidence of displacement of private units by public units in departments. If we view the number of public units as exogenous, 10 more public units are associated to 2.5 fewer private units. This finding supports hypothesis #2. Similarly, results from column (2) suggest that if we view the number of public units as exogenous, 10 more public units are associated to 1.8 fewer private units in markets of birth quantile 1 and 5, and to 2.5 fewer private units in markets of birth quantile 2, 3, and 4. The correlation across departments is consistent with the hypothesis that the number of births required to sustain a private unit is constant across markets and that an additional public maternity unit captures enough births to displace an almost constant number of private units across departments.

As reported in column (3) of Table 3, our instrumental variable approach suggests some evidence of displacement of private by public units in departments. A lower number of public units increases the number of private units across all market sizes. On average 10 more public units are associated to 2.8 less private maternity units. Column (4) reports the point estimates of the effect of public units on private units across market sizes. These correspond to the point estimates estimated in column (2). However, the effects across birth quantiles are highly imprecise for inference.

### 5.2. Reduced-Form Analysis on Intensive Margin Substitution

In this subsection we introduce the reduced-form analysis of substitution between private and public hospitals. As the analysis is carried out from the hospital’s point of view, we first illustrate how we defined each hospital’s competitor and then provide the empirical strategy followed by the results.

#### 5.2.1. Maternity Units Market Definition

To define a market, two main elements are required: Product definition and geographic definition. As we are only dealing with maternity units, the product definition is trivial. However, as patients are assumed to minimize their travel distances, a geographical market definition is required. We expect patients to be heterogeneous in their traveling preferences, leading to markedly different geographical markets across regions [3,31,32].

We use a catchment area analysis to define geographical markets. We do so by computing the driving time required from the user’s to the unit’s location, as defined by the centroids of the zip-codes. The geographical market is defined by a catchment area around the hospital (from now on, the focal unit) from which 95% of patients originate. The area is defined by an imaginary circle that has as its center the location of the focal unit. The 95% is a threshold value that can be allowed to vary. We vary the size of the threshold from 75%, 80%, and 99%. We assume that the catchment area does not change across time. Moreover, to construct the catchment area we use information on all years. As we construct a distance-weighted competition proxy, our findings are robust to the choice of the threshold. The hospital’s catchment area can be understood as the distance (in traveling time) that the majority of patients (i.e., say a threshold 95% of patients) are willing to travel. Any public or private hospital whose catchment areas intersect are considered to exert a competitive constraint on the focal unit. Using driving time, rather than a fixed radius, reduces the likelihood of having distorted geographical markets due to population density [3]. There are several precedents of use of isochrones as proxies for geographical markets. In France, the relevant geographical markets are defined as local markets within a radius of 30 min driving time. The radius may be extended up to one hour depending on the type of care, the population density, and the available hospital infrastructure [8]. In the UK, merger investigations used isochrones of 30 min defined by having 80% of private hospital patients come from areas within a 30-min drive-time (Office of Fair Trading, 2008a; Office of Fair Trading, 2008b). Isochrone analysis has also been used for defining geographical markets in the NHS hospital mergers. All hospitals within a 30–40 min drive-time were considered as belonging to the same geographical market. To construct the maternity unit *j*’s market share, we divide the number of births occurring in a maternity unit *j* by the total number of births occurring in its catchment area. We are able to know the number of birth per zip-code per year. Thus, we sum the births over the zip-codes that belong to each maternity units’ catchment area.

#### 5.2.2. Empirical Specification

To assess the substituability between private and public maternity units, we regress the natural logarithm of the focal unit market share on the natural logarithm of the number of for-profit, non-for-profit, and public hospitals located within the market of each focal unit. To account for the competitive pressure between hospitals, we use the distance-to-hospital weighed number of competitors by applying as weights the inverse of the distance to the focal unit. This implies that the further away a competitor is located, the smaller the competitive pressure to the focal unit. In a separate specification, we also control for the size and distance to the focal unit. We measure the size of a hospital using the number of beds. Our findings are robust to this re-weighting.

We distinguish the impact of the number of public, for-profit, and non-for-profit units in the markets on private units’ market shares and control for unit- and time-fixed effects. The specification is as follows:

(7) $$log\left(s_{jt}\right)=\beta_{0}+\beta_{1}log\left(FP_{jt}\right)+\beta_{2}log\left(NP_{jt}\right)+\beta_{3}log\left(PU_{jt}\right)+\gamma_{t}+\gamma_{j}+\alpha_{1}X_{jt}+\epsilon_{jt},$$

where $log(s_{jt})$ is the natural logarithm of the market share of the maternity unit *j* in year *t*; FP*_j__t_* captures the distance-to-hospital weighted number of for-profit units in maternity unit *j*’s catchment area and in year *t*; and NP*_j__t_* and PU*_j__t_* are, respectively the distance-to-hospital weighted number of a non-for-profit status and public maternity units in maternity unit *j*’s catchment area and in year *t*; and $X_{jt}$ are time-varying controls specific to unit *j*, as reported in Table 1. Finally, $\gamma_{t}$, $\gamma_{j}$, and $\epsilon_{jt}$ capture time-fixed effects, unit-fixed effects, and unit-time idiosyncratic variation, respectively.

In addition, we also distinguish the impact of the competitive pressure depending on the population size (measured as the number of births) of the department. The specification is as follows:

(8) $$\begin{array}{cc} log\left(s_{jt}\right)=\beta_{0}+ & \sum_{k=1}^{5}\beta_{1k}log\left(FP_{jt}\right)\times BQ_{jt}^{k}+\sum_{k=1}^{5}\beta_{2k}log\left(NP_{jt}\right)\times BQ_{jt}^{k}+\\ \; & \sum_{k=1}^{5}\beta_{3k}log\left(PU_{jt}\right)\times BQ_{jt}^{k}+\gamma_{t}+\gamma_{j}+\alpha_{1}X_{jt}+\epsilon_{jt}, \end{array}$$

where $BQ_{mt}^{j}$ is a dummy variable that equals one if the market of the focal unit, *m*, is located within a department that is considered as belonging to the *j*th quantile in the French birth population distribution at year *t*, and zero otherwise.

Our identification strategy entails potential endogeneity issues. That is, unobserved market characteristics might influence both the maternity units’ market shares and the number of competing maternity units. For example, if users strongly prefer public provision, more public units will be present and private units will have smaller market shares. The estimated relation between the number of public and private units’ market shares not only captures the substitution patterns between the two types of units, but it also encompasses future users’ preferences for public units, if such were to be the case. To try to correct these potential endogeneity sources, we control for hospital fixed effects. In doing so, our identifying variation is solely derived from changes in the number of competitors over time. Therefore, as long as hospital environments, including households’ tastes, do not change over time, we control for these sources of endogeneity.

To try to control for the remaining potential endogeneity, we propose an instrumental variable approach. To instrument for the number of competing maternity units, we propose to exploit the time variation in the potential market that each competitor faces. As a large number of births is likely to sustain a greater number of maternity units, the potential market that each unit faces serves as a good predictor of the number of maternity units. Thus, for each competitor we compute the number of births it faces within a radius of 50 km across the years. Then, similar to the variable capturing the number of competitors, we normalize the potential market using the distance to the focal hospital. That is, we construct a distance-to-hospital weighted average of hospital *j*’s competitors’ potential market.

#### 5.2.3. Results

We report the estimated coefficients for the model assessing the potential substitution between maternity units. The potential substitution for private units (Table 4), and for public units (Table 5) are reported separately. An observation is a combination of a given year and a given focal maternity unit. The dependent variable in all regressions is the natural logarithm of the focal unit’s market share, taking the relevant market of the unit. Independent variables are the natural logarithm of the number of other units in the focal unit’s relevant market, as well as the focal unit’s observed time-varying characteristics. Unit- and time-fixed effects are introduced in all regressions. For the sequence of results’ presentation, we consider results regarding within sector competition (private to private and public to public) and between sectors competition (private to public and public to private) successively.

Considering private sector competition, results indicate that a focal unit’s market share decreases with the number of other private for-profit units (columns (1) and (2) of Table 4). The magnitude of the point estimate increases when maternity units are introduced (columns (2) of Table 4) and persists after the use of the potential market instrument (column (4) of Table 4). The decrease is observed mostly in high-birth departments (column (3) of Table 4). Private for-profit units significantly affect private focal units’ market shares, with an increase of 10% in the number of private units estimated to decrease a private focal unit market share by 3.50%. Market shares remain unaffected by the number of other private maternity units when private focal units are located in low-birth departments, indicating that there may be no between competition in the private sector in those departments. Nevertheless, the corresponding IV point estimates (Column (5) of Table 4) suggest a decrease in low-birth departments, but they are imprecisely estimated. In higher-birth departments, however, results suggest that an increase of 10% in the number of private competitor units decreases market shares by at least 2.8%.

Considering public sector competition, results suggest that a public focal unit’s market share decreases with the number of other public units, indicating within public competition (columns (1) and (2) of Table 5). The magnitude of the point estimate doubles after use of the potential market instrument (column (4) of Table 5) but the coefficient is less precisely estimated. Public units significantly affect public focal unit’s market shares, with an increase of 10% in the number of public units estimated to decrease a public focal unit’s market share by 7.28%. The point estimates remain quite consistent across birth quantiles but the effect is only statistically significant in departments with the highest birth quantile. In the highest-birth departments, results suggest that an increase of 10% in the number of public competitor units decreases market shares by at least 16.5%.

Regarding sector competition, we first consider the impact of public units on private market shares (Table 4). We find that, on average, the market shares of private units decrease with the number of public units. All the point estimates across specifications are negative, but only the specification with fixed effects produces effects that are not statistically significant. The specification with the potential market instrumental variable yields a coefficient that is more than twice the size as the specification without fixed effects but it is less precisely estimated. The point estimate suggests that an increase of 10% in the number of public units decreases the market shares by 6.77%. The point estimates are quite consistent across focal units located in different birth size departments. The effect is statistically significant for market shares of private focal units located in quintiles 2, 3, and 5 birth departments. An increase by 10% of the number of public units in these markets decreases private market shares by no less than to 10%. Turning to the impact of private units on public market shares (Table 5), we find that public market shares are not affected by the number of private units. Introducing the potential market instruments increases the size of the estimated coefficients but they remain relatively small and imprecisely estimated. These results suggests that competition is tough within sectors and in an highly asymmetric way across sectors. Thus, this finding supports hypothesis # 3.

## 6. Discussion and Conclusions

In price-regulated systems where hospitals compete on the basis of quality, it is generally acknowledged that competition increases quality. The extent of the competitive pressure exerted by firms on one another, however, depends on their substituability. In this paper, we sought to disentangle the degree of substituability between private and public providers in the French maternity market. By focusing on maternity units where user choice is central, we investigated the potential for displacement between public and private maternity units in two levels. First, we assessed the degree of substitution both in the number of maternity units (i.e., on the extensive-margin) and second we assessed it in users’ choices.

To provide these contributions, we built over a stylized economic model of hospital competition. Next, to consistently estimate the competitive effects of public versus private maternity units, we constructed an original data set on obstetric services for public and private hospitals in France. With it, we empirically tested the following three hypotheses. First, we hypothesized that given a softer budget constraint, the number of public maternity units should be much larger in less populated departments than in more populated ones. In line with hypothesis #1, we observed a negative relationship between the birth quantiles and the number of public maternity units. Second, we established that public maternity units may displace private maternity units. Our results indicated that there was evidence of displacement of private units by public units in departments. If we view the number of public units as exogenous, 10 more public units are associated to fewer units by 2.5 private units less. This finding supports hypothesis #2. Finally, we examined the extent and nature of competition between existing producers. Our results suggest that competition is tough within sectors and in a highly asymmetric way across sectors. The results showed that an increase of 10% in the number of public units decreased the market shares by about 6.8% while public market shares did not seem to be affected by the number of private units. Thus, this finding supports hypothesis #3. In short, as users choice in the maternity market is free, the patterns that we identify in the paper suggest that users may, on average, prefer public over private providers. Thus, exit of private providers may not hurt user welfare.

Our research contributes to identifying the potential consequences of private/public hospital closures on hospital services’ availability. For example, in recent years, governments have increasingly opened up health care markets to private for-profit providers. In Norway, the health care market for psychiatric care and substance abuse treatment was opened up to private for-profit providers in November 2015. More recently, German Länders have transferred a large part of hospital activity to the private sector. Finding out whether a reduction/increase in public service provision will lead the private sector to enter/exit the market is therefore an important question that has not yet been documented extensively so far. More importantly, the consequences on users’ welfare is not accounted for. To the extent that a user prefers a type of provider over another, not having access to their preferred provider may be detrimental to their welfare.

Our paper focuses on maternity care and further research is needed in order to extend this substitution analysis to other healthcare services. To extent of our knowledge, this is one of the first contributions on the substituability between for-private and public providers in the maternity unit market. Identifying services for which substitution between private and public provision may (or not) apply is essential for both decision-makers in charge of planning public hospital service provision and for private providers decision to enter the market.

The strengths of our approach are twofold: First, the choice of a rather unique and under-researched institutional context, i.e., French maternity units, where competition between public and private units is sufficiently developed for substitution analyses to be meaningful and second, the quality of our merged data set, which enables us to control for risk and quality heterogeneity, in order to compare public and private maternity units.

Our study has limitations. Our analysis, like most of the international research that exploits catchment areas, relies on a simple definition of the maternity units catchment area [33]. Even if robustness checks on the definition of the catchment area do not significantly affect our findings, we could still not be capturing the true catchment area of each maternity unit. Another limitation is potential endogeneity issues, as unobserved market characteristics might influence both maternity units’ numbers and market shares as well as the number of competing maternity units. Units’ fixed effects partly control for this but we must still rely on the assumption that units’ environments do not change in different ways over time for different units. Our reduced-form results are likely to be underestimating the effects on the intensive and extensive margins of substitution. To correct for these issues, we use instrumental variables that yield potentially convincing results (the size of the coefficients increase in the expected direction) but still rely on strong identification assumptions.

## Figures and Tables

**Table 1:** Maternity units summary statistics.

Variables	Mean	Std Dev.	Min	Max
**# of births per department**	12,825	9301	651	36,703
**Hospital characteristics**			
Relative length of stay	0.994	0.0736	0.452	1.390
C-section rate (in %)	19.98	4.238	6.260	46.45
Epidural rate (in %)	74.07	14.46	0.130	98.90
Births per obstetrician	49.55	90.32	1	4840
Mid-wife per obstetrician	3.786	1.792	0.200	21.30
Bed utilization rate	59.08	15.47	0.200	319.4
Number of beds	42.01	50.28	3	1077
Number of scanners	1.786	1.987	0	43
Number of MRI	1.894	1.644	0	34
Certification level 2	0.0287	0.167	0	1
Certification level 3	0.172	0.378	0	1
Certification level 4	0.131	0.337	0	1
Certification level 5	0.279	0.449	0	1
**# of for-profit maternity units**			
Level 1	1.805	1.717	0	6
Level 2	0.816	0.976	0	4
All	2.622	2.224	0	8
**# of non-for-profit maternity units**			
Level 1	0.369	0.756	0	3
Level 2	0.161	0.426	0	2
All	0.531	0.898	0	4
**# of public maternity units**			
Level 1	1.564	1.399	0	6
Level 2	1.991	1.504	0	7
Level 3	0.795	0.735	0	3
All	4.349	2.592	1	14
**Patients characteristic**			
Driving time to maternity unit (mins)	21.91	20.98	2.06	130.37

**Table 2:** Number of public and private maternity units (2009–2014).

Panel A: Public	All	Level 1	Level 2	Level 3
Year				
2009	340	128	149	63
2010	334	123	148	63
2011	332	121	148	63
2012	329	118	148	63
2013	328	114	151	63
2014	327	113	151	63
**Panel B: Private for-profit**	All	Level 1	Level 2	Level 3
Year				
2009	165	117	48	0
2010	154	108	46	0
2011	149	103	46	0
2012	144	98	46	0
2013	142	95	47	0
2014	140	90	50	0
**Panel C: Private non-for-profit**	All	Level 1	Level 2	Level 3
Year				
2009	32	24	8	0
2010	31	23	8	0
2011	31	23	8	0
2012	30	22	8	0
2013	29	21	8	0
2014	29	21	8	0

**Table 3:** Extensive margin substitution between maternity units

	(1)	(2)	(3)	(4)
	FE	FE	FE-IV	FE-IV
# public maternity unit	−0.245 **		−0.279 *	
	(0.0989)		(0.151)	
birth quintile 1 x # public maternity unit		−0.191 **		−0.311
		(0.0861)		(0.306)
birth quintile 2 x # public maternity unit		−0.259 **		−0.335
		(0.104)		(0.272)
birth quintile 3 x # public maternity unit		−0.263 **		−0.327
		(0.102)		(0.293)
birth quintile 4 x # public maternity unit		−0.281 ***		−0.219
		(0.101)		(0.336)
birth quintile 5 x # public maternity unit		−0.187 *		−0.0391
		(0.106)		(1.357)
Observations	564	564	564	564
R-squared	0.340	0.305	-	-
Number of departments	94	94	94	94
Department FE	YES	YES	YES	YES
Year FE	YES	YES	YES	YES

*Notes:* Dependent variable is the number of maternity units per department-year. Each column represents a different regression focusing on a subset of maternity units. The subset for each column is indicated by the column header. Standard errors are clustered at the department level. The Cragg–Donald Wald F statistic is 29.069 and the Hansen J statistic’s *p*-value is 0.127. Column (4) standard errors are department-level bootstrap standard errors (using 100 replications).

**Table 4:** Reduced Analysis on substitution between maternity units: Focal unit is private

	FE			FE-IV	
	(1)	(2)	(3)	(4)	(5)
Logarithm number of FP	−0.160 **	−0.338 ***		−0.350 ***	
	(0.0625)	(0.0976)		(0.0736)	
x birth quintile 1			0.0384		−0.143
			(0.311)		(0.307)
x birth quintile 2			−0.162		−0.200
			(0.198)		(0.239)
x birth quintile 3			−0.270 ***		−0.286 **
			(0.0605)		(0.133)
x birth quintile 4			−0.452 ***		−0.405 **
			(0.129)		(0.170)
x birth quintile 5			−0.624 ***		−0.692 ***
			(0.228)		(0.261)
Logarithm number of PU	−0.241 **	−0.129		−0.677*	
	(0.105)	(0.136)		(0.402)	
x birth quintile 1			−1.082 ***		−0.995
			(0.321)		(0.807)
x birth quintile 2			−0.909 ***		−1.037 *
			(0.218)		(0.544)
x birth quintile 3			−0.815 ***		−1.067 **
			(0.268)		(0.537)
x birth quintile 4			0.238		−0.682
			(0.200)		(0.481)
x birth quintile 5			−0.000690		−1.112 **
			(0.0280)		(0.507)
Logarithm number of NFP	−0.178 **	−0.0572 *		−0.0634 **	
	(0.0712)	(0.0309)		(0.0278)	
x birth quintile 1			−0.218		−0.0851
			(0.207)		(0.431)
x birth quintile 2			−0.100		−0.0913
			(0.134)		(0.187)
x birth quintile 3			−0.0726 ***		−0.0926 *
			(0.0238)		(0.0545)
x birth quintile 4			−0.105 ***		−0.100
			(0.0297)		(0.0651)
x birth quintile 5			−0.205 **		−0.157
			(0.0922)		(0.145)
Observations	774	774	774	774	774
R-squared	0.675	0.97	0.97		
Hospital FE	NO	YES	YES	YES	YES
Department FE	YES	YES	YES	YES	YES
Year FE	YES	YES	YES	YES	YES
Number of id		145	145	145	145

*Notes:* Dependent variable is the logarithm of hospitals’ market share as defined by their catchment area. The logarithm # of FP, PU, and NFP are all weighted by their corresponding distance to the focal unit. Hospitals with only one competitor in either category are not considered in this analysis. Standard errors are clustered at the department level.

**Table 5:** Reduced analysis on substitution between maternity units: Focal unit is public

	FE			FE-IV	
	(1)	(2)	(3)	(4)	(5)
Logarithm number of FP	0.0277	−0.0288		−0.0579	
	(0.0418)	(0.0479)		(0.0428)	
x birth quintile 1			0.0804		0.0550
			(0.0546)		(0.0593)
x birth quintile 2			0.0233		−0.000854
			(0.0528)		(0.0624)
x birth quintile 3			−0.0360		−0.0809
			(0.0468)		(0.0503)
x birth quintile 4			−0.0477		−0.103 **
			(0.0424)		(0.0508)
x birth quintile 5			−0.0251		−0.109
			(0.0573)		(0.118)
Logarithm number of PU	−0.669 ***	−0.311 **		−0.728 *	
	(0.112)	(0.137)		(0.434)	
x birth quintile 1			−0.392 **		−0.706
			(0.176)		(0.521)
x birth quintile 2			−0.238 **		−0.693
			(0.114)		(0.454)
x birth quintile 3			−0.212 *		−0.663
			(0.115)		(0.484)
x birth quintile 4			−0.186		−0.568
			(0.118)		(0.472)
x birth quintile 5			−0.375 **		−1.655 ***
			(0.177)		(0.575)
Logarithm number of NFP	−0.00317	−0.0901 **		−0.110 ***	
	(0.0499)	(0.0384)		(0.0263)	
x birth quintile 1			−0.0543 **		−0.116 ***
			(0.0267)		(0.0411)
x birth quintile 2			−0.0796 **		−0.112 **
			(0.0332)		(0.0529)
x birth quintile 3			−0.0628 **		−0.0969 **
			(0.0303)		(0.0443)
x birth quintile 4			−0.0811 **		−0.130 **
			(0.0370)		(0.0529)
x birth quintile 5			−0.0981 **		0.104
			(0.0378)		(0.0848)
Observations	1707	1707	1707	1707	1707
R-squared	0.531	0.94	0.94		
Hospital FE	NO	YES	YES	YES	YES
Department FE	YES	YES	YES	YES	YES
Year FE	YES	YES	YES	YES	YES
Number of id		293	293	293	293

*Notes:* Dependent variable is the logarithm of hospitals’ market share as defined by their catchment area. The logarithm # of FP, PU, and NFP are all weighted by their corresponding distance to the focal unit. Hospitals with only one competitor in either category are not considered in this analysis. Standard errors are clustered at the department level.
